# Cyr61和WISP-3在非小细胞肺癌组织中的表达及其临床意义

**DOI:** 10.3779/j.issn.1009-3419.2010.12.08

**Published:** 2010-12-20

**Authors:** 建发 吕, 友成 邹, 纯伟 张, 志福 毛

**Affiliations:** 1 431600 汉川，湖北省汉川市人民医院胸外科 Department of Toracic Surgery, People's Hospital of Hanchuan, Hanchuan 431600, China; 2 430060 武汉，武汉大学人民医院胸外科 Department of Toracic Surgery, Renmin Hospital of Wuhan University, Wuhan 430060, China

**Keywords:** Cyr61, WISP-3, 肺肿瘤, 免疫组织化学, Cyr61, WISP-3, Lung neoplasms, Immunohistochemisty

## Abstract

**背景与目的:**

*Cyr61*是非小细胞肺癌（non-small cell lung cancer, NSCLC）生长过程中的一个肿瘤抑制基因，*Cyr61*与*WISP-3*同属于CCN基因家族，具有极其明显的序列同源性。本研究通过检测Cyr61和WISP-3在NSCLC组织中的表达，探讨其临床意义。

**方法:**

应用免疫组化SP染色法检测54例NSCLC癌组织和癌旁正常肺组织中Cyr61和WISP-3的表达，并结合临床参数进行分析。

**结果:**

在NSCLC癌组织中Cyr61表达水平低于癌旁正常肺组织（*P* < 0.001），WISP-3表达水平高于癌旁正常肺组织（*P* < 0.001）；NSCLC癌组织中Cyr61与WISP-3蛋白表达呈负相关（*r*=-0.395, *P*=0.003）；Cyr61的表达与肿瘤的组织学分化程度、病理类型、临床分期、家族史、吸烟史和淋巴结转移有关（*P* < 0.05）；WISP-3阳性表达率与肿瘤的组织学分化程度、临床分期和年龄有关（*P* < 0.05）。

**结论:**

Cyr61和WISP-3可能是反映NSCLC进展、生物学行为、转移发生及预后的重要生物学标记物。

## Abstract (en)

**objective:**

Cysteine-rich protein 61 (Cyr61) plays a role as a tumor suppressor in non-small cell lung cancer (NSCLC). Cyr61 and WISP-3 have a very significant sequence homology, belonging to the same CCN gene family. The aim of this study is to investigate the expressions of Cyr61 and WISP-3 in NSCLC, and explore the relationship between their expressions and tumor's clinicopathological characteristics.

**Methods:**

The expressions of Cyr61 and WISP-3 were detected in 54 cases with primary NSCLC and their corresponding normal lung tissues in control group by immunohistochemical staining (SP), and the clinical data were analyzed.

**Results:**

Down-regulation of Cyr61 and up-regulation of WISP-3 were both found in lung cancer tissue compared with the corresponding normal lung tissue (both *P* < 0.001); The expression of Cyr61 was negatively correlated with the expression of WISP-3 (*r*=-0.395, *P*=0.003); Cyr61 expression levels was closely correlated with tumor grade, tumor type, clinical stage, family history, smoking and metastasis (*P* < 0.05). Also, WISP-3 was closely correlated with tumor grade, clinical stage and age (*P* < 0.05).

**Conclusion:**

The expressions of Cyr61 and/or WISP-3 may be important biological markers in reflecting the progression, biological behaviors, metastatic potential and prognosis of NSCLC.Background and

至今，已知CCN家族有6个成员，分别是Cyr61/ CCN1（cystein-rich 61）、结缔组织生长因子（CTGF/ CCN2）、肾母细胞瘤过度表达基因（*NOV/CCN3*）、WNT诱导的分泌型蛋白-1（WISP-1/CCN4）、WISP-2/ CCN5和WISP-3/CCN6，CCN家族是根据该家族中最初发现的3个成员Cyr61、CTGF和NOV的首字母命名的。

*Cyr61*被称为潜在的肿瘤抑制基因，可抑制非小细胞肺癌（non-small cell lung cancer, NSCLC）肿瘤细胞的生长、浸润、转移^[1]^；*WISP-3*是CCN基因家族的一个新成员，有关其生物学特征的报道较少。近年来研究发现，*WISP-3*基因与某些肿瘤的发生发展有着密切关系，因此其成为CCN家族的研究热点之一。本研究采用免疫组织化学方法对NSCLC标本中Cyr61和WISP-3表达进行检测，并探讨其临床意义。

## 1 资料与方法

### 1.1 一般资料

武汉大学人民医院胸外科2008年9月-2009年10月临床确诊的NSCLC癌组织（NSCLC组）及其相应的癌旁正常肺组织（对照组）标本各54例。患者男性41例，女性13例，年龄43岁-74岁，中位年龄56.2岁；病理类型：鳞癌32例，腺癌17例，腺鳞癌3例，细支气管肺泡癌2例（编为“其它”）；肿瘤细胞的组织学分化程度分为高分化（Ⅰ级）、中度分化（Ⅱ级）和低分化（Ⅲ级），组织学分级：Ⅰ级9例，Ⅱ级27例，Ⅲ级18例；临床分期：Ⅰ-Ⅱ期21例，Ⅲ期-Ⅳ期33例。有淋巴结转移者35例，无淋巴结转移者19例。所有病例术前均未行放化疗，并且均有完整的临床资料、病理分级。

### 1.2 主要试剂

多克隆抗体兔抗人Cyr61（1:1 000）、羊抗人WISP-3（1:1 000）、SP试剂盒（通用型）均购自美国Chemicon国际有限公司。

### 1.3 实验方法

所有组织标本均经10%福尔马林固定、脱水、石蜡包埋，切成4 μm厚切片，采用免疫组化SP染色法检测NSCLC癌组织中Cyr61及WISP-3的表达情况。实验中以PBS代替一抗作为阴性对照，用已知的阳性NSCLC切片作为阳性对照，操作步骤按SP试剂盒说明书进行。

### 1.4 免疫组织化学染色结果判断

以细胞浆呈不均匀棕黄色细颗粒状、网状或棕褐色团块状为阳性染色。本研究采用了既包括染色强度又包括染色范围的4级计分系统以判断染色结果^[2]^。每张切片在光镜下随机选取5个高倍视野，每个视野计数100个瘤细胞（异型增生）。根据染色强度计算：无染色0分，浅黄色为1分，棕黄色为2分，棕褐色为3分；染色强度需与背景着色相对比。根据染色肿瘤细胞数占肿瘤细胞总数的比例计算： < 10%为1分；10%-50%为2分； > 50%为3分。再将上述两种计分相乘，总分≤3分定义为基因表达明显减少或缺乏（即阴性表达）；总分 > 3分则被定义为基因表达阳性。所有的切片染色结果均由一位病理科医生和作者共同作出判断。

### 1.5 统计学方法

应用SPSS 13.0统计软件包进行*χ*^2^检验，两变量间相关分析采用*Spearman*等级相关分析，以*P* < 0.05为差异具有统计学意义。

## 2 结果

### 2.1 Cyr61和WISP-3在NSCLC和正常肺组织中的表达情况

Cyr61主要在正常肺组织细胞质和细胞间隙中表达，可见很强的Cyr61蛋白表达（图 1A），而NSCLC癌组织中表达相对很弱（图 1B）；正常肺组织中Cyr61的阳性表达率为88.89%，NSCLC组织中Cyr61的阳性表达率为48.15%，差异具有统计学意义（*χ*^2^=20.776, *P* < 0.001）。与Cyr61表达的结果相反，WISP-3主要在癌组织细胞浆中表达，NSCLC患者的癌组织切片中可见很强的WISP-3表达（图 2A），而在癌旁正常肺组织切片中表达相对较弱（图 2B）；9例正常肺组织表达WISP-3（16.67%），36例NSCLC癌组织切片表达WISP-3（66.67%），差异具有统计学意义（*χ*^2^=33.75, *P* < 0.001）（表 1）。

**1:**
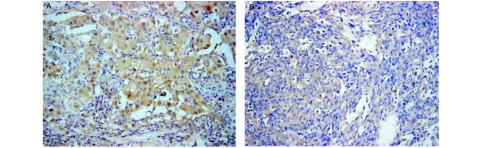
Cyr61在正常肺组织及非小细胞肺癌组织中的阳性表达。A：Cyr61在正常肺组织中阳性表达（SP，×200）；B：Cyr61在非小细胞肺癌组织中阳性表达（SP，×200）。 Expression of Cyr61 in normal lung tissue and NSCLC tissue. A: Expression of Cyr61 in normal lung tissue (SP, ×200); B: Expression of Cyr61 in NSCLC tissue (SP, ×200).

**2:**
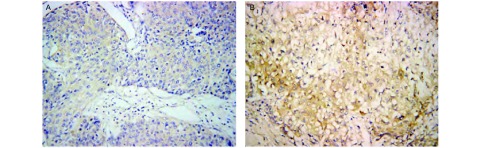
WISP-3在正常肺组织及非小细胞肺癌组织中的阳性表达。A：WISP-3在正常肺组织中阳性表达（SP，×200）；B：WISP-3在非小细胞肺癌组织中阳性表达（SP，×200）。 Expression of Cyr61 in normal lung tissue and NSCLC tissue. A: Expression of WISP-3 in normal lung tissue (SP, ×200); B: Expression of WISP-3 in NSCLC tissue (SP, ×200).

**1:** Cyr61和WISP-3在NSCLC和正常肺组织中的表达情况 Expressions of Cyr61 or WISP-3 in NSCLC and normal lung tissue

Group	*n*	Cyr61					WISP-3			
		(-)	(+)	%	*P*		(-)	(+)	%	*P*
NSCLC	54	28	26	48.15	< 0.001		18	36	66.67	< 0.001
Normal	54	6	48	88.89			45	9	16.67	

### 2.2 Cyr61和WISP-3蛋白表达的相关性

在NSCLC组中，Cyr61与WISP-3表达均阳性者14例，均阴性者3例；WISP-3阳性、Cyr61阴性者25例；WISP-3阴性、Cyr61阳性者12例，经*Spearman*等级相关分析显示两者呈负相关（*r*=-0.395, *P*=0.003）（表 2）。

**2:** Cyr61、WISP-3在NSCLC中表达的相互关系 The relationship between the expressions of Cyr61 and WISP-3 in NSCLC

	Cyr61		*r*	*P*
	(+)	(-)		
WISP-3			-0.395	0.003
(+)	14	25		
(-)	12	3		

### 2.3 Cyr61和WISP-3表达与NSCLC临床病理参数之间的关系

Cyr61的阳性表达与肿瘤的组织学分化程度、病理类型、临床分期、家族史、吸烟史和淋巴结转移有关（*P* < 0.05），与年龄、性别、肺结核病史和肿块大体类型无关（*P* > 0.05）。Cyr61的阳性表达率随组织学分化程度的升高而升高，高-中分化组阳性率（61.11%）明显高于低分化组（22.22%）（*P*=0.007）；随临床分期的升高而下降，临床Ⅲ期-Ⅳ期表达（33.33%）明显低于Ⅰ期-Ⅱ期（71.43%），差异有统计学意义（*P*=0.006）。

鳞癌组Cyr61表达的阳性率（62.5%）明显高于腺癌组（29.41%）（*P*=0.027）；有家族史组Cyr61表达的阳性率（16.67%）明显低于无家族史组（57.14%）（*P* =0.013）；淋巴结转移阳性组C y r61的表达率（25.71%）明显低于淋巴结转移阴性组（78.95%）（*P* < 0.001）；吸烟史组Cyr61表达率（32.35%）明显低于无吸烟史组（75%）（*P*=0.002）。WISP-3阳性表达率与肿瘤的组织学分化程度、临床分期和年龄有关（*P* < 0.01），而与其它临床指标无关（*P* > 0.05）。WISP-3的阳性表达率随组织学分化程度的升高而降低，低分化组阳性率（94.44%）明显高于高中分化组（61.11%）（*P*=0.01）；随临床分期的升高而升高，临床Ⅲ期-Ⅳ期表达率（84.85%）明显高于Ⅰ期-Ⅱ期（52.38%），差异有统计学意义（*P*=0.009）；≥60岁组WISP-3的阳性表达率（90%）明显高于 < 60岁组阳性表达率（50%）（*P*=0.001）（表 3）。

**3:** Cyr61和WISP-3表达与NSCLC临床病理参数之间的关系 The relationship between the expression Cyr61 and WISP-3 and the clinical parameters of NSCLC

Factor	*n*	Cyr61		*χ*^2^	*P*	WISP-3		*χ*^2^	*P*
		(+)	%			(+)	%		
Age				1.962	0.161			10.630	0.001^☆☆^
< 60	24	9	37.50			12	50.00		
≥60	30	17	56.67			27	90.00		
Gender				2.071	0.150			2.882	0.090
Male	41	22	53.37			32	78.05		
Female	13	4	30.77			7	53.85		
Family history				6.125	0.013^☆^			0.059	0.808
Yes	12	2	16.67			9	75.00		
No	42	24	57.14			30	71.43		
Tuberculosis				0.775	0.379			0.036	0.849
Yes	8	5	62.5			6	75.00		
No	46	21	45.65			33	71.74		
Smoking				9.174	0.002^☆☆^			0.826	0.363
Yes	34	11	32.35			26	76.47		
No	20	15	75.00			13	65.00		
Pathology					0.027^☆☆^				0.559^*^
SCC	32	20	62.50			25	78.12		
ADC	17	5	29.41			12	70.58		
ASC	3	1	33.33			1	33.33		
Other	2	0	0			1	50.00		
Tumor stage				7.269	0.007^☆☆^			6.646	0.010^☆☆^
Ⅰ-Ⅱ	36	22	61.11			22	61.11		
Ⅲ	18	4	22.22			17	94.44		
Clinical stage				7.460	0.006^☆☆^			6.743	0.009^☆☆^
Ⅰ-Ⅱ	21	15	71.43			11	52.38		
Ⅲ-Ⅳ	33	n	33.33			28	84.85		
Metastasis				14.130	< 0.001^☆☆^			1.201	0.273
Yes	35	9	25.71			27	77.14		
No	19	15	78.95			12	63.16		
^☆^: *P* < 0.05; ^☆☆^: *P*≤0.01; ^*^: Difference between squamous cell carcinoma and adenocarcinoma; SCC: Squamous cell carcinoma; ADC: Adenocarcinoma; ASC: Adenosquamous carcinoma.									

## 3 讨论

本研究显示，癌组织中Cyr61表达水平低于癌旁正常肺组织，提示Cyr61在NSCLC的发生发展中可能是抑癌基因；相反，癌旁正常肺组织中WISP-3表达水平明显低于癌组织，推测WISP-3可能是NSCLC的致癌基因。有报道指出，*Cyr61*基因对多种组织的肿瘤细胞起调控作用。人胰腺癌腹膜转移癌的癌组织中Cyr61高表达^[3]^；卵巢癌Cyr61能够刺激细胞增殖并抑制细胞凋亡，在其发生过程中起重要作用^[4]^。有文献^[1, 5]^报道，*Cyr61*是NSCLC生长过程中的一个肿瘤抑制基因，Cyr61能够促使p53上调，而后引发一系列的信号转导，阻滞细胞周期的G_1_期。Gery等^[4]^通过转染实验证实：以不含内源性*Cyr61*基因的NSCLC细胞株为实验材料，用Cyr61的cDNA表达子转染的NSCLC细胞株表达的克隆数比用相同数量的阴性表达子转染的NSCLC细胞株表达的克隆数少60%-90%。WISP-3已被证实与肿瘤生长有关，在不同类型的肿瘤中可能起着正向调节或负向调节肿瘤生长的作用。Kleer等^[6, 7]^研究发现，在炎性乳腺癌组*WISP-3*基因是一种抑癌基因，具有抑制肿瘤细胞增殖、侵犯周边组织、血管生成及促进细胞分化的作用。另有研究结果^[8-11]^表明，在结直肠癌、异常微卫星不稳定性胃癌、肝细胞癌的研究中发现WISP-3在这些肿瘤组织中呈高表达或突变状态。Davies等^[12]^研究表明，在乳腺癌组织中*WISP-1*是一种抑癌基因，WISP-2为一种肿瘤刺激因子，而WISP-3与肿瘤的发生与发展无确切关系。

统计分析显示，临床参数对Cyr61及WISP-3表达有重要影响。统计分析显示，病理组织学低分化组的Cyr61表达水平明显低于高分化组，临床分期晚组明显低于分期早组。Yu等^[13]^也报道在病理组织学分化低的软骨肉瘤中低表达Cyr61，这与NSCLC中的情况是一致的。Cyr61的阳性表达与家族史、吸烟史和淋巴结转移有关，与Cui等^[14]^的研究相一致。有淋巴结转移的肿瘤中Cyr61表达量低于无淋巴结转移的肿瘤，这些结果提示Cyr61可能是反映NSCLC生物学行为和预后的重要标记物。WISP-3阳性表达率与肿瘤的组织学分化程度、临床分期和年龄等有关。根据癌组织和癌旁正常组织中的表达水平可以证实，对于肺癌来说，*Cyr61*是抑癌基因而*WISP-3*是致癌基因。可见*Cyr61*和*WISP-3*单个基因或两者相互作用对肺癌的发展起重要作用，其表达的蛋白产物可作为临床干预治疗的重要靶蛋白。对CCN与肿瘤关系的进一步研究和分子机理的深入阐明将会对肿瘤的早期诊断、治疗和预防提供理论依据。

本研究显示，在NSCLC组织中Cyr61和WISP-3蛋白表达呈密切负相关（*r*=-0.395, *P*=0.003），提示两者之间的恒定比例和一定表达水平对正常肺组织的维持非常重要，Cyr61和WISP-3的表达失衡在NSCLC发生和发展过程中起重要作用，其作用机制有待进一步探讨。
